# Relationship between Latitude and Melanoma in Italy

**DOI:** 10.5402/2012/864680

**Published:** 2012-01-16

**Authors:** Emanuele Crocetti, Carlotta Buzzoni, Alessandra Chiarugi, Paolo Nardini, Nicola Pimpinelli

**Affiliations:** ^1^Clinical and Descriptive Epidemiology Unit, Institute for Cancer Study and Prevention (ISPO), Via delle Oblate 2, 50141 Florence, Italy; ^2^Airtum Databank, c/o Clinical and Descriptive Epidemiology, Unit Institute for Cancer Study and Prevention (ISPO), Via delle Oblate 2, 50141 Florence, Italy; ^3^Melanoma Early Diagnosis Service, Institute for Cancer Study and Prevention (ISPO), Via Cosimo il Vecchio 2, 50141 Florence, Italy; ^4^Department of Dermatological Sciences, University of Florence Medical School, Piazza Indipendenza 11, 50121 Florence, Italy

## Abstract

*Objective*. Evaluate the ecological relationship between skin melanoma epidemiology and latitude in Italy. *Methods*. We used data from the Italian network of cancer registries (Airtum). In a Poisson model, we evaluated the effect on incidence, mortality, and survival of latitude, adjusting for some demographic, social, phenotypic, and behavioural variables. *Results*. Incidence increased in Italy by 17% for each degree of increase in latitude. The effect of latitude was statistically significantly present also adjusting for other variables (incidence rate ratio = 1.08). The effect of latitude on increasing mortality (mortality rate ratio = 1.27) and improving survival (relative excess risk of death = 0.93) was no longer present in the multivariate model. *Conclusion*. Melanoma incidence, mortality, and survival vary in Italy according to latitude. After adjustment for several confounders, incidence still grows with growing latitude. Presumably, latitude expresses other variables that might be related to individual susceptibility and/or local care.

## 1. Introduction

Although Italy is “the land where the lemon trees bloom,” as Goethe recited [1], the Italian climate changes dramatically from the cold Alpine regions in the North, to the subtropical areas in the South [2]. Italy stretches for about 1,000 kilometres from 47° to 35° latitude north, and the ultra violet (UV) irradiation varies accordingly with a north to south increasing trend [3]. 

Sun exposure is included among the major causes of skin melanoma (MM) [4] although its role is still controversial [5]. The UV affects skin causing genetic changes and immune function impairment; it also stimulates the production of growth factors and the formation of free radicals [6]. Phenotype traits significantly influenced the risk of melanoma [7].

The incidence of MM in white populations generally increases when latitude decreases, with the highest recorded incidence occurring in Australia, where the annual rates are 10–20 times the rates in Europe [8]. In New Zealand, MM incidence and mortality rates increased with increasing proximity to the equator in both sexes [9].

A Norwegian study described a latitude gradient for MM with decreasing incidence with increasing latitude [9, 10]; the same result was found in Sweden, for both general population [11] and children [12], and in Australia [13]. MM incidence increased at lower latitude [14] also among non-Hispanic whites in the USA. Studies in Spain [15], in Sweden [16], and in Australia and New Zeeland [17] document a link between latitude and MM mortality. In the USA, an upward gradient of MM mortality from north to south was documented in the past [18]. 

In Sweden, the increase in sun exposure (moving from north to south) may lead to improved prognosis for several cancers [9]. Also in Norway cancer patients resident in regions with high UV showed a better prognosis than those living in low UV regions probably related to higher calcidiol concentrations [19].

In Italy, MM incidence, mortality, and survival vary greatly across the country [20–22].

The present study evaluates the ecological relationship between MM figures and latitude in Italy.

## 2. Materials and Methods

In Italy, there are several regional population-based cancer registries included in the Italian Network of Cancer registries (AIRTUM). Cancer registration started in the late 1970s and has since progressed and incorporating wider areas across the Country. There are currently 30 general cancer registries and 5 specialized ones (by age or cancer site). Overall about 22,000,000 people, more than 33% of the Italian population, are monitored for cancer incidence (http://www.registri-tumori.it).

For each cancer registry, latitude has been defined as the latitude of the main town of the registration area as reported on Google Earth 6.0.2. (http://earth.google.com/intl/it/).

The registries included in our study vary from 46°30*'* (Bolzano) to 36°53*'* (Ragusa) latitude north. We used published incidence and mortality data for the period 1998–2002 from 20 cancer registries [20]. As regards survival, we analysed 5-year relative survival rates for cancers incident during 1995–1999 [21].

The relationship between incidence and mortality and latitude has been evaluated by means a Poisson model, which includes the number of cancer cases (or cancer deaths) as well as latitude, sex, and age (0–44, 45–59, 60–74, and 75+ years).

Moreover, we included in the model an economic variable: the mean income (in 2002) for each province where a registry is active or the mean for many provinces if more than one were included in the registration area. 

We express this data as index numbers, with the Italian mean being 100 [23]. This economic variable ranged among the analysed areas from 64.3 to 152.8 (although the southern provinces were consistently below the Italian mean). Finally, we included among the variables in the model also the proclivity to cancer prevention, that is, the mean regional percentage of asymptomatic women who stated to have had a mammography in 2000.

To estimate the effect of pigmentary traits, we included the mean proportion of resident in the Italian regions with blond hair; this is from a historical estimate according to the conclusion presented in the pivotal research conducted by Ridolfo Livi during army medicals in 1859–1863 [24]. To our knowledge, no updated data is available on phenotype or on single pigmentary traits.

As regard the relationship between latitude and survival, we used a generalized linear model with Poisson error on aggregated data that includes the same variables of the models for incidence and mortality [25]. We computed also Relative Excess Risk of death (RER).

We evaluated the effect of each variable in improving the multivariate models by means of the likelihood ratio tests. As regards survival, we included annual time since diagnosis in each fitted model but did not report it in the result tables.

Our research includes the computation of the linear correlation between latitude and variables under study.

## 3. Results

The incidence rate ratio (IRR) increased in Italy by about 17% for each degree of latitude (Table 1). Most of the other analysed variables were correlated with latitude: the mean provincial income (linear correlation 0.80), the frequency of mammography (0.84), and the proportion of blond hair (0.72). The multivariable Poisson model that best fits the data (pseudo *R* ^2^ = 0.694)—which includes age, mean provincial income, proportion of blond hair residents, and latitude—shows that the number of cases increases with age and income—when the proportion of blond subjects is higher—and with latitude, at a rate of 8% for each degree of latitude (IRR = 1.082, 95% CI 1.054–1.111), (Table 1). Sex did not show a statistically significant effect in the univariate analysis and did not improve the multivariate model. MM incidence increased by about 3% for every unit of percent increase in mammography attendance, but this variable also did not improve the multivariate model.

The mortality rate ratio (MRR) (Table 2) is higher with ageing and in male subjects, who have 27% more increased risk than women; it is also higher with the increase in the index of mean provincial income, when the proclivity to cancer prevention is higher, when the proportion of blond residents is higher, and when latitude increases (MRR = 1.115).

In the multivariate model, the inclusion of latitude and mammography did not improve the fit of the model, Table 2.

Relative survival seems to be related with latitude, with a statistically significant decrease by about 7% of the relative excess risk of death (relative hazard ratio) for each further degree of latitude. The excess risk of dying decreases where the provincial income is higher and the mammography testing increases. We could detect no relationship between the risk of dying and the mean proportion of blond residents (Table 3).

Latitude did not seem to improve the multivariate model that fitted at best the data, Table 3.

## 4. Discussion

The Italian network of cancer registries documented that MM incidence, mortality, and survival varied in Italy with higher rates in northern and central regions and lower rates in the southern ones [20–22].

On the contrary, it is widely documented that MM incidence and mortality increase as latitude approaches the equator [10, 12–18, 26]. Moreover, also survival seemed to have the same relationship with latitude [9, 19].

The effect of latitude may be correlated with the UV irradiation or of the relationship between UV and phototype but, also, with geographical differences in the local health system (e.g., diagnostic aggressiveness, quality of care, etc.) and with individual susceptibility or behaviour.

A recent case control study, including 5700 MM cases, showed a complex relationship between MM risk and patterns of sun exposure (recreational/occupational), body sites, sunburns, and latitude [27].

We tried to disentangle the role of latitude from the role possibly played by other variables in this ecological descriptive study, adjusting for some possible confounders.

We evidenced that in Italy latitude has a strong correlation with the mean provincial income, the proclivity to cancer prevention, and the proportion of blonde residents.

In the univariate analysis, the increase in latitude (moving from south to north) is significantly related with the increase in incidence, mortality, and survival for MM.

As regards incidence, such effect is halved but still present after adjustment for several confounders, such as age, mean provincial income, and proportion of blonde residents.

This means that geographical differences in age distribution, mean provincial income, and the proportion of blonde residents, although relevant in modifying the risk of MM incidence [28], do not explain completely the effect of latitude. The proclivity to mammographic screening, although significant in the univariate analysis, does not improve the multivariate model.

It is worthwhile to mention that the proportion of blonde residents describes a historical situation referring to about 150 years ago. Nowadays, a more homogeneous composition is expected due to internal and external migration. With a more updated variables—unfortunately not available yet—the effect of latitude would be presumably widened.

MM mortality increased with latitude but its effect disappeared once the role of other confounders was considered.

Latitude influences also survival: relative excess risk of death for MM was 7% lower for each degree of increase in latitude. This effect seems to be completely explained by the different geographical distribution of the other confounders, and it is no longer significant in the multivariate analysis.

Only sparse data are available regarding MM thickness in Italy. It shows that data incidence has grown especially for thin lesions [29]. The residual effect of latitude on incidence may be due to a higher skin preventive activity and skin bioptic aggressiveness in central and northern regions than in the south. Preventive activity and higher bioptic rates drive to higher proportion of thin lesions and to the diagnosis of indolent lesions [30]. If this were the reason for the higher incidence in northern Italy than in the south, we should also expect a latitude effect on survival. On the contrary, we did not found it in the multivariate model. Therefore, other explanations for such differences should be found.

MM incidence, mortality, and survival vary in Italy according to latitude. After adjustment for several confounders, the effect of latitude is still present as incidence grows with growing latitude. Such result does not seem related to a different early diagnosis activity. Presumably, latitude expresses other constitutional, behavioural, and environmental variables we had not included in the model and that might be related to individual susceptibility and/or local care.

## Figures and Tables

**Table 1:** Crude and adjusted Incidence Rate Ratio (IRR) and 95% confidence intervals for malignant melanoma for selected variables.

Variable	Crude IRR, 95% CI		Adjusted^*⋀*^ IRR, 95% CI	
Sex				
Female	1.000			
Male	0.956	0.912–1.001		
Age (years)				
0–44	1.000		1.000	
45–59	2.883	2.703–3.076	2.832	2.655–3.021
60–74	3.743	3.515–3.985	3.673	3.449–3.911
75+	4.339	4.041–4.658	4.218	3.929–4.529
Mean provincial Income 2002	1.011	1.010–1.013	1.004	1.003–1.006
Mammography 2000 (%)	1.026	1.023–1.029		
Blond hair 1859–63 (%)	1.040	1.036–1.045	1.014	1.007–1.020
Latitude (increasing degrees)	1.170	1.153–1.186	1.082	1.054–1.111

^*⋀*^Multivariate Poisson model including age, mean provincial 2002 income, % blond hair, and latitude. LR chi2(6) = 3074.41. Pseudo *R* ^2^ = 0.6942.

**Table 2:** Crude and adjusted Mortality Rate Ratio (MRR) and 95% confidence intervals for malignant melanoma for selected variables.

Variable	Crude MRR, 95% CI		Adjusted^*⋀*^ MRR, 95% CI	
Sex				
Female	1.000		1.000	
Male	1.276	1.154–1.410	1.587	1.434–1.755
Age (years)				
0–44	1.000		1.000	
45–59	4.707	3.893–5.690	4.699	3.887–5.681
60–74	9.855	8.289–11.716	9.993	8.405–11.882
75+	19.792	16.672–23.495	21.024	17.695–24.979
Mean provincial Income 2002	1.008	1.006–1.011	1.004	1.002–1.007
Mammography 2000 (%)	1.019	1.013–1.025		
Blond hair 1859–63 (%)	1.032	1.023–1.041	1.025	1.014–1.035
Latitude (increasing degrees)	1.115	1.083–1.147		

^*⋀*^Multivariate Poisson model including sex, age, mean provincial 2002 income, and % blond hair. LR chi2(6) = 1844.91. Pseudo *R* ^2^ = 0.7621.

**Table 3:** Relative survival: Relative Excess Risk of death (RER) and 95% confidence intervals for malignant melanoma for selected variables.

Variable	RER*, 95% CI		Adjusted^*⋀*^ RER*, 95% CI	
Sex				
Female	1.000		1.000	
Male	1.829	1.584–2.110	1.734	1.523–1.973
Age (years)				
0–44	1.000		1.000	
45–59	1.531	1.263–1.856	1.480	1.223–1.791
60–74	2.413	2.003–2.908	2.339	1.945–2.813
75+	4.006	3.237–4.957	4.101	3.317–5.069
Mean provincial Income 2002	0.990	0.985–0.994	0.991	0.987–0.994
Mammography 2000 (%)	0.989	0.983–0.994		
Blond hair 1859–63 (%)	0.994	0.981–1.007		
Latitude (increasing degrees)	0.931	0.895–0.969		

*Estimated relative excess risk of death by Generalized Liner Model on Relative Survival.

^*⋀*^Generalized Liner Model on Relative Survival including annul follow-up time (omitted in the table), sex, age, and mean provincial 2002 income.
